# Complete Genome Sequence of Salmonella enterica Serovar Typhimurium Myophage Mutine

**DOI:** 10.1128/MRA.00401-19

**Published:** 2019-05-09

**Authors:** Jamie Gutierrez, Yicheng Xie, Jason J. Gill, Mei Liu

**Affiliations:** a Center for Phage Technology, Texas A&M University, College Station, Texas, USA; Queens College

## Abstract

Mutine is a myophage of Salmonella enterica serovar Typhimurium. Here, we present the complete genome of Mutine (161,502 bp) and show that it is similar to that of phage Vi01.

## ANNOUNCEMENT

Salmonella spp. are major agents causing foodborne illnesses that result in at least 400 deaths per year (1). Strains of Salmonella enterica serovar Typhimurium are rapidly gaining antibiotic resistance in both developed and developing countries (2). The study of *S.* Typhimurium phages may offer alternative control means for this pathogen.

Phage Mutine, using *S.* Typhimurium as the host, was isolated from mixed municipal wastewater collected in Brazos County, TX, in 2016. Host bacteria were cultured on tryptic soy broth or agar (Difco) at 37°C with aeration, and phage isolation and propagation were done using the soft agar overlay method (3). Phage genomic DNA was prepared using a modified Promega Wizard DNA cleanup kit protocol, as previously described (4). Pooled indexed DNA libraries were prepared using the Illumina TruSeq Nano low-throughput (LT) kit, and the sequence was obtained using the Illumina MiSeq platform and the MiSeq v2 500-cycle reagent kit, following the manufacturer’s instructions, producing 538,626 paired-end reads for the index containing the phage genome. The reads were verified in FastQC 0.11.5 (https://www.bioinformatics.babraham.ac.uk/projects/fastqc/), trimmed with FastX-Toolkit 0.0.14 (http://hannonlab.cshl.edu/fastx_toolkit/), and built in SPAdes 3.5.0 (5). The genome was closed by PCR using primers 5′-AACACGCTGGGCATACTT-3′ and 5′-CGTCAACAAACACCCTCATTAC-3′ facing towards each end of the assembled contig and by Sanger sequencing of the resulting product, with the contig sequence manually corrected to match the resulting Sanger sequencing read. The genes that coded for proteins were predicted by the software GLIMMER 3.0 (6) and MetaGeneAnnotator 1.0 (7), along with manual correction; the tRNA genes were found using ARAGORN 2.36 (8). To predict the protein functions, BLASTp 2.2.28 (9) was used to find sequence homology, and conserved domain searches were found using InterProScan 5.15-5.40 (10). All analyses were done using default settings via the CPT Galaxy (11) and Web Apollo (12) interfaces (cpt.tamu.edu).

The Mutine genome was assembled into a contig of 161,502 bp with 153-fold coverage. There are in total 218 protein-encoding genes and 3 tRNAs in the Mutine genome. It has a GC content of 44.3% and a coding density of 92.9%. According to a BLASTp search at an expected (E) value of <10^−5^, Mutine shares 204 similar proteins with Vi01 (GenBank accession number FQ312032) (13), a member of a group of large myophages of the genus *Viunalikevirus* that are distantly related to phage T4 (14). Using the progressiveMAUVE algorithm (version 2.4.0) (15), Mutine shares 87% DNA similarity to Escherichia phage vB_EcoM_CBA120 (GenBank accession number NC_016570), another Vi01-like phage. The Mutine genome is presented as syntenic (in the same gene order) to phage T4, with antiabortive infection genes (RIIA or RIIB) at each end of the genome. Mutine has many expected morphogenesis proteins of Vi01-like phages, including a cluster of tail spike proteins, an arrangement which might lead to the umbrella-like structures of the tail (13, 14). A superinfection exclusion protein similar to phage P22 gp17 was identified (16). An *N*-acetylmuramidase-like endolysin is identified in Mutine, but no holins or spanin complex could be reliably identified. The tape measure protein was identified without an identifiable chaperone protein.

### Data availability.

The genome sequence of phage Mutine was deposited under GenBank accession number MG428992. The associated BioProject, SRA, and BioSample accession numbers are PRJNA222858, SRR8788533, and SAMN11260686, respectively.
